# Environmental interactions are regulated by temperature in *Burkholderia seminalis* TC3.4.2R3

**DOI:** 10.1038/s41598-019-41778-x

**Published:** 2019-04-02

**Authors:** Priscila Jane Romano de Oliveira Gonçalves, Carmen C. Denman Hume, Almir José Ferreira, Sarina Tsui, Marcelo Brocchi, Brendan W. Wren, Welington Luiz Araujo

**Affiliations:** 1 University of São Paulo, Department of Microbiology, São Paulo, 05508-900 Brazil; 2 London School of Hygiene and Tropical Medicine, Department of Pathogen Molecular Biology, London, WC1E 7HT United Kingdom; 3 University of Campinas, Department of Genetics, Evolution, Microbiology and Immunology, São Paulo, 13083-862 Brazil

## Abstract

*Burkholderia seminalis* strain TC3.4.2R3 is an endophytic bacterium isolated from sugarcane roots that produces antimicrobial compounds, facilitating its ability to act as a biocontrol agent against phytopathogenic bacteria. In this study, we investigated the thermoregulation of *B. seminalis* TC3.4.2R3 at 28 °C (environmental stimulus) and 37 °C (host-associated stimulus) at the transcriptional and phenotypic levels. The production of biofilms and exopolysaccharides such as capsular polysaccharides and the biocontrol of phytopathogenic fungi were enhanced at 28 °C. At 37 °C, several metabolic pathways were activated, particularly those implicated in energy production, stress responses and the biosynthesis of transporters. Motility, growth and virulence in the *Galleria mellonella* larvae infection model were more significant at 37 °C. Our data suggest that the regulation of capsule expression could be important in virulence against *G. mellonella* larvae at 37 °C. In contrast, *B. seminalis* TC3.4.2R3 failed to cause death in infected BALB/c mice, even at an infective dose of 10^7^ CFU.mL^−1^. We conclude that temperature drives the regulation of gene expression in *B. seminalis* during its interactions with the environment.

## Introduction

*Burkholderia* species are widely distributed in the environment, including soil^1^, water^2^, plant tissues^3^, several animal species^4^ and in humans, particularly in hospital settings^5^. Members of the *B. cepacia* complex (Bcc) have been isolated as opportunistic pathogens that cause lung infections in immunocompromised patients with cystic fibrosis^6^. However, many species from this Bcc group have been isolated from plants and possess the ability to control plant diseases^7^ or to promote plant growth^8^. Root colonization ability does not depend on source or species in the Bcc group^9^, suggesting that members of the Bcc group can differentially regulate genes depending on their interactions with plant or animal hosts or in the soil environment where they compete with other microbial species.

Among *Burkholderia* species, *Burkholderia seminalis* was recently described^10^ and included in the Bcc group. This gram-negative, aerobic, non-sporulating, rod-shaped bacterium forms yellow-pigmented mucoid colonies on agar plates. *B. seminalis* has been found in several habitats, including water^11^, soil^12^, plants^7^ and the sputum of cystic fibrosis patients^1^. In plants, *B. seminalis* may be associated with growth promotion^12^, biocontrol of orchid necrosis^7^, root nodulation^13^ or phytopathogenesis, specifically peach rot^14^.

*B. seminalis* strain TC3.4.2R3 was isolated endophytically from sugarcane roots^15^ and was shown to be effective in the biocontrol of orchid necrosis caused by *B. gladioli*^7^. The TC3.4.2R3 strain produces pyochelin, a rhamnolipid, and other unidentified diffusible metabolites that have been associated with inhibition of *Fusarium oxysporum*^16^ and the cacao pathogens *Moniliophthora perniciosa* (fungus), *Phytophthora citrophtora, P. capsici*, and *P. palmivora* (oomycete)^17^. Therefore, *Burkholderia* spp. are considered effective biological control agents because they produce an array of antimicrobial compounds^18^. Several metabolites, including cepacins, pyrrolnitrins, cepaciamides, cepacidines, quinolones, phenazines, siderophores, lipopeptides, among others^19,20^ have been reported for *Burkholderia* species, and most of them present antifungal activity.

Many crops are infected by phytopathogenic fungi, and some of them, such as *Fusarium oxysporum*, may cause rot and vascular wilt in several cultures, including onion, tomato, lettuce, cotton, and brassicas, among others^21,22^. *Colletotrichum* spp. are other important phytopathogens that damage fruits and vegetables by causing blight and anthracnose spots in addition to postharvest rot^23^. Pineapple disease, wilt and postharvest black rot are plant diseases caused by *Ceratocystis fimbriata* and *Ceratocystis paradoxa* in economically important crops such as sugarcane^24,25^. Therefore, the use of *Burkholderia* species as a biocontrol agent may prevent economic losses in a diverse range of crops with important global implications. To date, the interactions of *B. seminalis* with its several hosts have been poorly studied.

Many Bcc members are human pathogens; therefore, testing the virulence of the TC3.4.2R3 strain is crucial to validate its possible use as an agricultural biocontrol agent. Larvae of the greater wax moth *Galleria mellonella* have been widely used as a non-mammalian model to investigate bacterial pathogenesis because the innate immune system of this insect is similar in many aspects to that of the mammalian innate system^26^. Comparison of bacterial pathogenicity in *G. mellonella* and mouse infection models is important to develop our understanding of the interactions of *B. seminalis* with different hosts.

Temperature is a central trigger for the control of gene expression in bacterial pathogens^27^. For example, differentially expressed virulence factors, such as toxins, are affected by temperature in *Pseudomonas syringae*^28^ and *Yersinia entomophaga*^29^. Motility, flagellar expression and biofilm production are often thermoregulated, for example, *P. syringae*^28^ and *Campylobacter jejuni*^30^. Furthermore, surfactant and exopolysaccharide (EPS) production appears to occur in a temperature-dependent manner in *Pseudomonas putida*^31^ and *Erwinia amylovora*^32^, respectively. Many genes involved in plant-microbe interactions^28^, insect-microbe interactions^29^, microbe-microbe interactions^33^ and biocontrol potential^34^ are also thermoregulated. Given that *B. seminalis* is a member of the Bcc that could have biocontrol applications, this study was undertaken to investigate the thermoregulation of *B. seminalis* strain TC3.4.2R3 in various hosts.

## Results

### Thermoregulation of biofilm formation, EPS and motility in *B. seminalis* TC3.4.2R3

**Figure 1:**
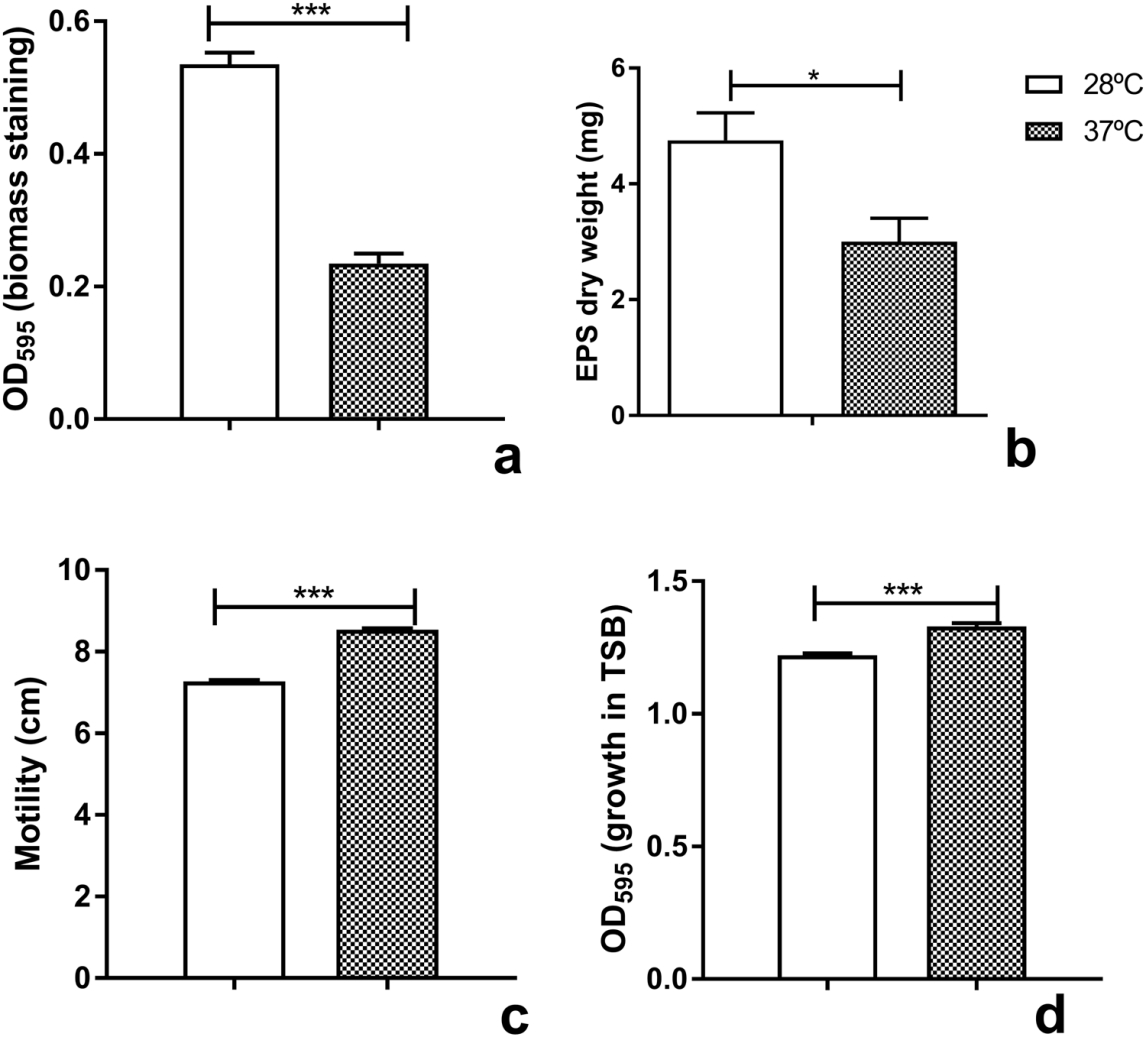
Comparative phenotypic profile of *B. seminalis* TC3.4.2R3 at 28 °C and at 37 °C. (**a**) Biofilm production; (**b**) EPS production in dry weight; (**c**) swimming motility; and (**d**) growth in TSB medium. For (**a**,**d**), the error bars represent the standard deviation of three technical replicates and eight biological replicates each; ***p < 0.0005. For (**b**), the error bars represent standard deviation; *p < 0.05 and (**c**) ***p < 0.0001.

To understand the role of temperature variation in strategies used by *B. seminalis* TC3.4.2R3 during ecological interactions, biofilm and EPS production, growth rate and swimming motility were evaluated (Fig. 1). We assessed the ability of *B. seminalis* TC3.4.2R3 to form biofilms using the MBEC (Minimum Biofilm Eradication Concentration) biofilm device of Innovotec Inc. (Edmonton, AB, Canada). Biofilm formation at 28 °C was 2.5-fold greater than that at 37 °C (Fig. 1a), and EPS production was 40% greater (*p* < 0.05) at 28 °C than at 37 °C (Fig. 1b). However, the swimming rate was significantly higher at 37 °C than at 28 °C (Fig. 1c, *p* < 0.0001), suggesting that biofilm production and motility are inversely related.

The growth rate was evaluated, and the highest rate occurred at 37 °C (Fig. 1d) at *p* < 0.0001. Growth curves were constructed for the TC3.4.2R3 strain at 28 °C and 37 °C based on the OD_595_ values recorded at 2 h intervals during incubation (Supplementary Fig. S1). Growth curve analysis revealed that *B. seminalis* exhibited the highest growth rate at 37 °C. The stationary phase was reached before 20 h of incubation for *B. seminalis* cultured at 37 °C and was delayed until 26 h of incubation at 28 °C, showing that after this time, both cultures were in stationary phase. A linear regression analysis was used to establish growth equations: Y = 0.2931*X − 0.4318 for growth at 28 °C and Y = 0.2889*X + 0.2895 for 37 °C. The results for best fit comparisons between data sets revealed that the curves were parallel with significantly different intercepts (*p* = 0.0470).

### Effect of temperature on antagonism against plant pathogenic fungi

**Figure 2:**
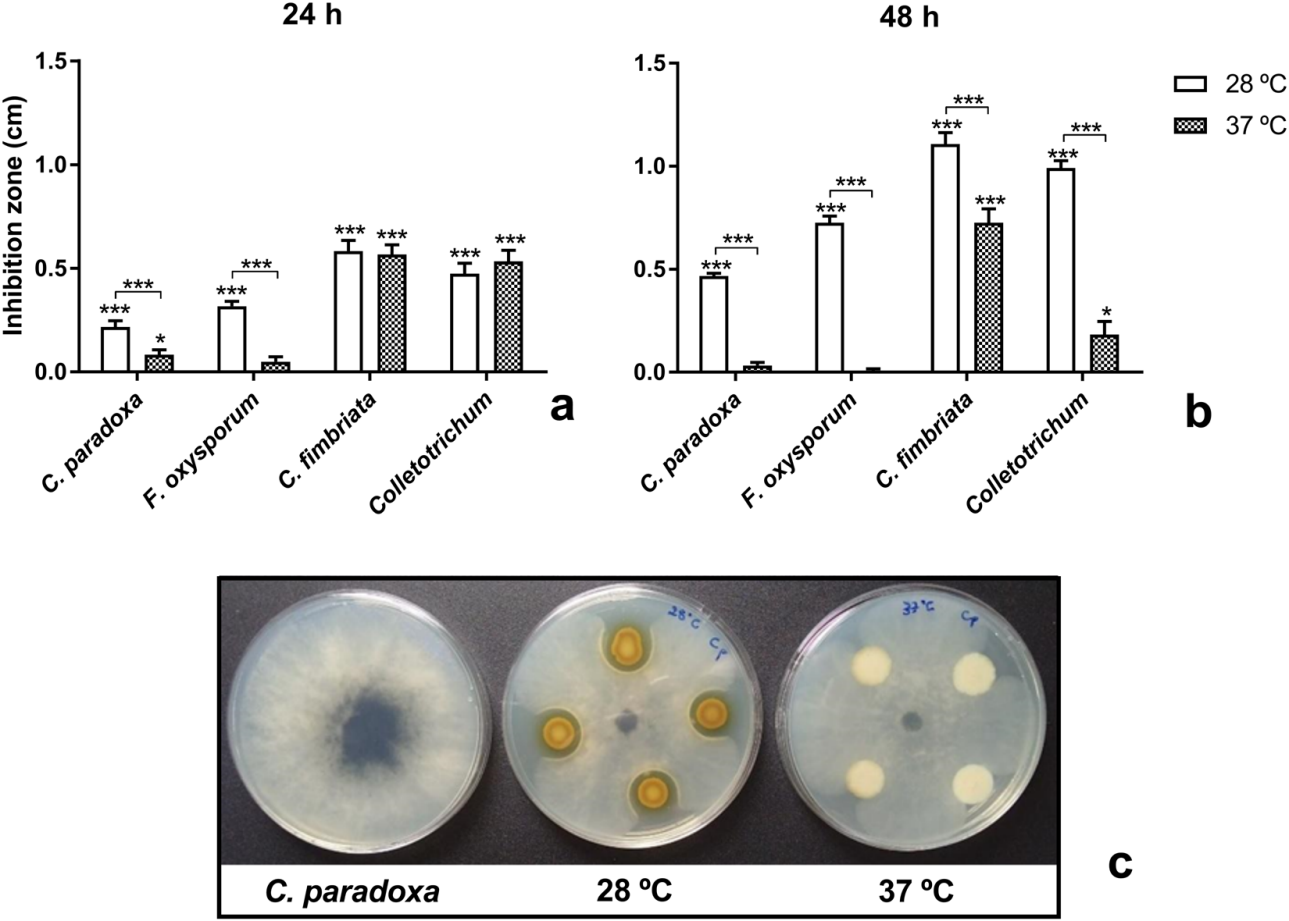
Antagonism of *B. seminalis* against phytopathogenic fungi at different temperatures. Bacteria were previously grown for (**a**) 24 h or (**b**) 48 h. The inhibition zone was measured in centimetres (cm). The error bars represent standard deviation; asterisks directly above columns indicate a significant difference compared to control plates; asterisks above lines indicate differences between temperature treatments: *p < 0.01 and ***p < 0.0001. (**c**) Antagonism of *B. seminalis* TC3.4.2R3 previously grown for 48 h at 28 °C and at 37 °C against *C. paradoxa*. Less yellow pigment was produced at 37 °C.

The ability of *B. seminalis* TC3.4.2R3 to inhibit the phytopathogenic fungi *F. oxysporum, C. paradoxa, C. fimbriata* and *Colletotrichum* spp. was evaluated at 28 °C and at 37 °C (Fig. 2). The ability of *B. seminalis* to inhibit *in vitro* phytopathogenic fungi was significantly higher at 37 °C than at 28 °C (Fig. 2a,b). In addition, we observed that *B. seminalis* produced less yellow pigment at 37 °C than at 28 °C (Fig. 2c) after 48 h of growth (Fig. 2a,b). Although fungal inhibition was observed when the bacteria were grown on plates for 24 h, the inhibition halo was twofold higher when grown for 48 h (Fig. 2a,b).

### Effects of *B. seminalis* TC3.4.2R3 on seed germination

To verify whether temperature influences the interaction of *B. seminalis* with a plant host, we investigated the effects of *B. seminalis* inoculation on seed germination at 28 °C and 37 °C in maize, a monocotyledonous, and cotton, a dicotyledonous species (Supplementary Table S1). Seed germination was not affected by TC3.4.2R3 at any temperature.

### Pathogenicity of *B. seminalis* TC3.4.2R3 infection is thermoregulated and host-dependent

**Figure 3:**
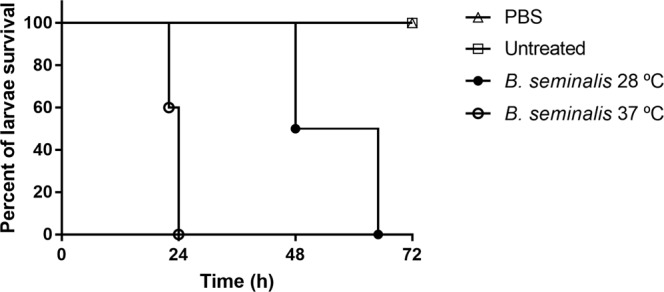
Demonstration of killing of *G. mellonella* larvae by *B. seminalis* TC3.4.2R3. Untreated: the larvae were not inoculated – line means the same results for both 28 °C and 37 °C; PBS: larvae inoculated with PBS - line means the same results for both 28 °C and 37 °C; *B. seminalis* 28 °C: larvae inoculated with *B. seminalis* and incubated at 28 °C; *B. seminalis* 37 °C: larvae inoculated with *B. seminalis* and incubated at 37 °C. Significance was determined using the log-rank (Mantel-Cox) test and Bonferroni correction; *p* < 0.0001.

The pathogenic potential of *B. seminalis* TC3.4.2R3 was evaluated in *G. mellonella* larvae (Fig. 3) and BALB/c mice. The *B. seminalis* TC3.4.2R3 pathogenic potential against *G. mellonella* was significant (*p* < 0.0001) and temperature-dependent. At 37 °C, by 24 h after inoculation with *B. seminalis*, all larvae had died. In contrast, at 28 °C, less virulence was observed; the larvae began to die after 48 h, and 100% death was observed after 65 h. In control experiments, no larvae death was observed for untreated larvae or larvae inoculated with PBS at both temperatures. Because strain TC3.4.2R3 showed higher virulence in the wax moth larvae infection model at 37 °C than at 28 °C, we proceeded to use the BALB/c mouse infection model to evaluate virulence in a mammalian model.

The pathogenicity of *B. seminalis* to BALB/c mice was evaluated up to 30 days with intraperitoneal inoculations of 10^7^, 10^6^, 10^5^, 10^4^ or 10^3^ CFU.mL^−1^ as the final concentration of bacteria. For all experiments, no signs of disease were observed, even at the highest bacterial inoculum.

### RNAseq analysis of *B. seminalis* TC3.4.2R3 under different temperatures

We used RNA sequencing and comparative transcriptomic analysis to identify genes and their respective expression levels at 28 °C and 37 °C in *B. seminalis* TC3.4.2R3 and to identify genes that potentially contribute to temperature and phenotype changes in *B. seminalis* TC3.4.2R3. Although we used environmental (28 °C) and clinical (37 °C) temperatures, the gene expression should be more related to antifungal production than to pathogenesis, since after 48 h of growth, the bacterial culture was in stationary phase. As previously presented, although there was fungal inhibition by *B. seminalis* grown for 24 h, the higher antifungal production occurred after 48 h of growth (Fig. 2b) when the 28 °C and 37 °C cultures were in stationary stage. As a consequence of these observations, RNAseq was performed at these time points and temperatures.

Through the generation of more than 35 million reads per sample, we were able to map approximately 2 Gb of the TC3.4.2R3 genome sequence to the corresponding sequences in the *B. seminalis* TC3.4.2R3 genome (GenBank Accession Number LAEU01000000) that includes three genetic elements: chromosome 1 (3.50 Mb), chromosome 2 (3.05 Mb) and a mega-plasmid (1.09 Mb). Table 1 displays the details of the yield and quality of Illumina sequencing per sample. The average depth was 235X coverage for 28 °C samples and 233X coverage for 37 °C samples.

**Table 1:** Transcriptome yield and quality.

Sample Name	PF* Yield (bp)	Number of reads	Q30%	Average Quality Score	Barcode	Depth coverage
PR-S1	1,825,288,292	35,789,967	93.32	37.26	TTAGGC	233
PR-S2	1,898,429,381	37,224,106	93.73	37.38	GATCAG	242
PR-S3	1,791,337,311	35,124,261	93.53	37.32	GTCCGC	229
PR-S4	1,863,094,566	36,531,266	93.66	37.36	CGTACG	238
PR-S5	1,862,172,843	36,513,193	93.75	37.38	ACTGAT	238
PR-S6	1,757,953,017	34,469,667	93.79	37.39	ATTCCT	225

Description: *PF: Passed Filter. PR-S1 to PR-S3: 28 °C triplicate samples. PR-S4 to PR-S6: 37 °C triplicate samples.

**Figure 4:**
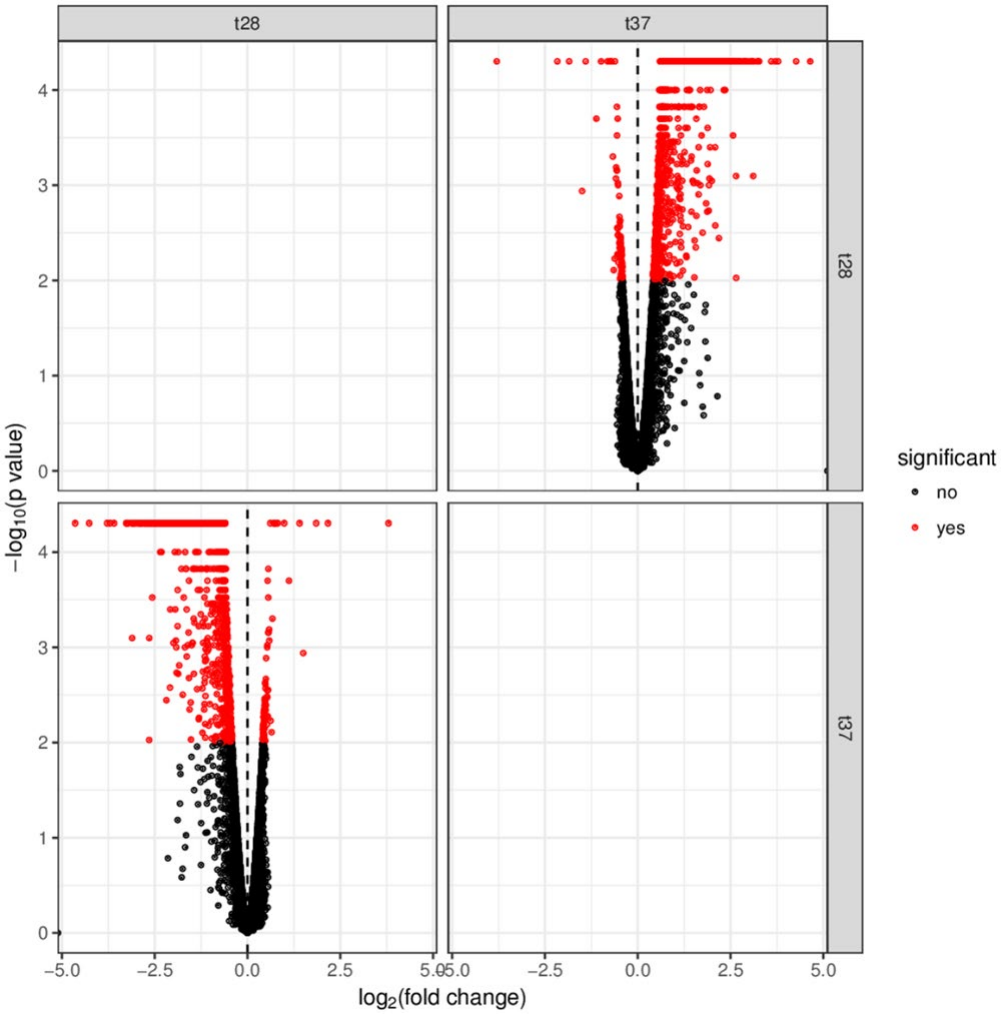
Results of the transcriptome analyses. Volcano scatter plot of log2 fold-change (FC) vs adjusted P-values (−log10) of *B. seminalis* grown at 28 °C vs 37 °C.

**Figure 5:**
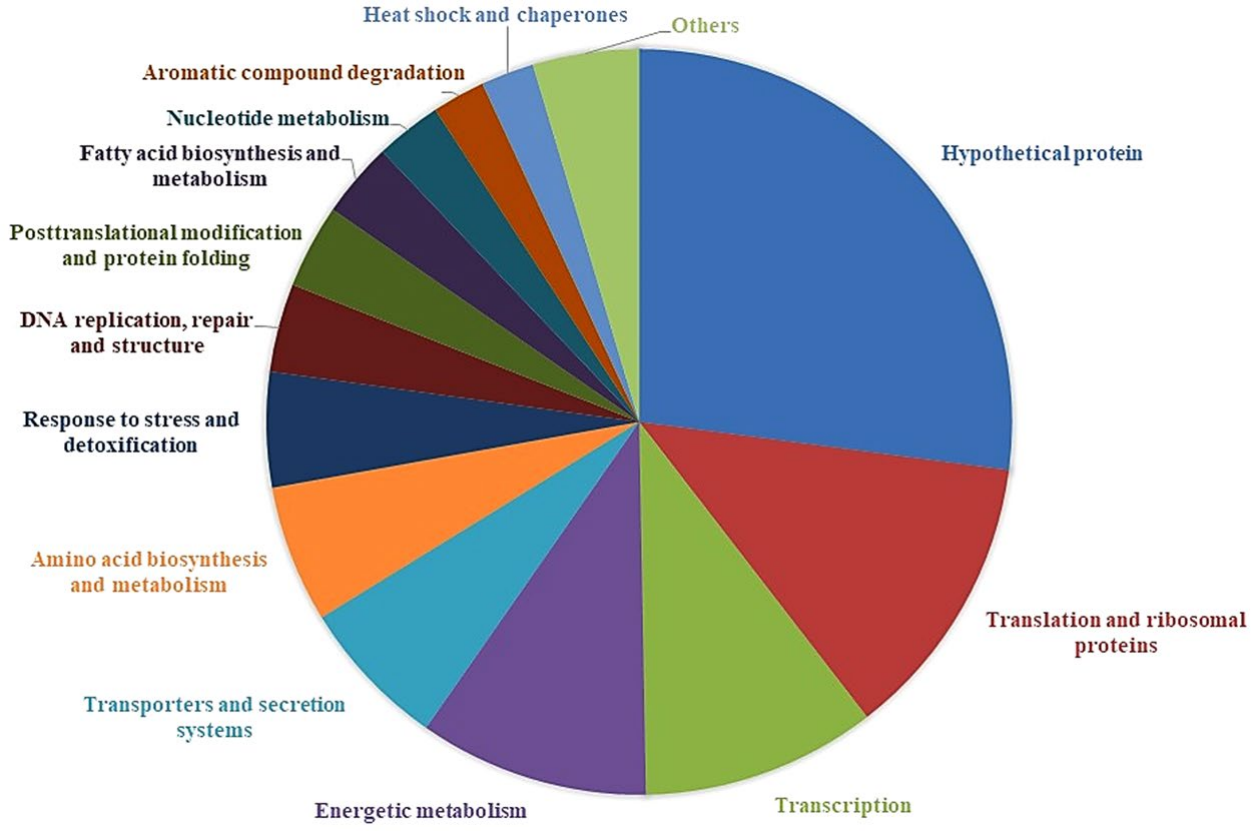
Distribution of induced genes. Functional classification of the 583 upregulated genes at 37 °C in *B. seminalis* TC3.4.2R3.

The RNA libraries were of high quality (Fig. 4), showing that 6912 (99.9%) of the 6917 genes were expressed. Only the pyochelin cluster was completely repressed at both 28 °C and 37 °C. To classify differentially expressed genes, we adopted a log2 fold-change (FC) above 1. Using this cutoff, 589 genes were differentially expressed, with 583 upregulated and six downregulated at 37 °C relative to 28 °C. Among these, 141 differentially regulated genes encoded hypothetical proteins, suggesting that these unknown genes could be related to adaptation to various environmental conditions. The downregulated genes encoded flagellin (*Bsem*_00099) and five hypothetical proteins (Supplementary Table S2). Functional classification of upregulated genes showed that these genes encode core functions such as energy metabolism, transport, regulatory proteins, cellular processes, and aromatic compound degradation and detoxification (Fig. 5).

The genes upregulated at 37 °C included 65 genes encoding translation and ribosomal proteins, 53 related to transcription regulation, 52 related to energy metabolism, 34 related to transport/secretion systems and 31 related to amino acid biosynthesis and metabolism. Growth at 37 °C also resulted in the upregulation of genes involved in responses to stress and detoxification (26 genes), DNA replication, repair and structure (20 genes), posttranslational modification and protein folding (19 genes), fatty acid biosynthesis and metabolism (15 genes), nucleotide biogenesis and metabolism (15 genes) and aromatic compound degradation (12 genes). Furthermore, 12 heat shock proteins and chaperones were upregulated at 37 °C, as well as genes encoding cofactors and vitamin metabolism (10 genes), terpenoids and polyketide metabolism (nine genes), virulence factors (eight genes), cold shock (six genes), signal transduction systems (six genes), toxin-antitoxin system and toxins (five genes), sulfur metabolism (four genes), flagella and pili (four genes), polyhydroxyalkanoate (PHA) biosynthesis (four genes), polysaccharide biosynthesis and metabolism (four genes), quorum-sensing (two genes) and lipopolysaccharide (LPS) biosynthesis (two genes) (Fig. 5). Furthermore, other genes, such as methyltransferases, cell division protein FtsB, GTP-binding proteins and AAA-ATPases, were also upregulated.

Genes encoding flagellin FliC (−2.17-fold) and the flagellar capping protein FliD (−0.72-fold) were downregulated at 37 °C. The flagellar transcriptional regulators FlhD and FlhC were upregulated (2.18- and 2.13-fold, respectively) at 37 °C, as was *fliL* (*Bsem*_03217), which encodes a flagellar basal body-associated protein and an OmpA/MotB domain-containing protein, which could be involved in forming the flagellar motor (Supplementary Table S3).

**Figure 6:**
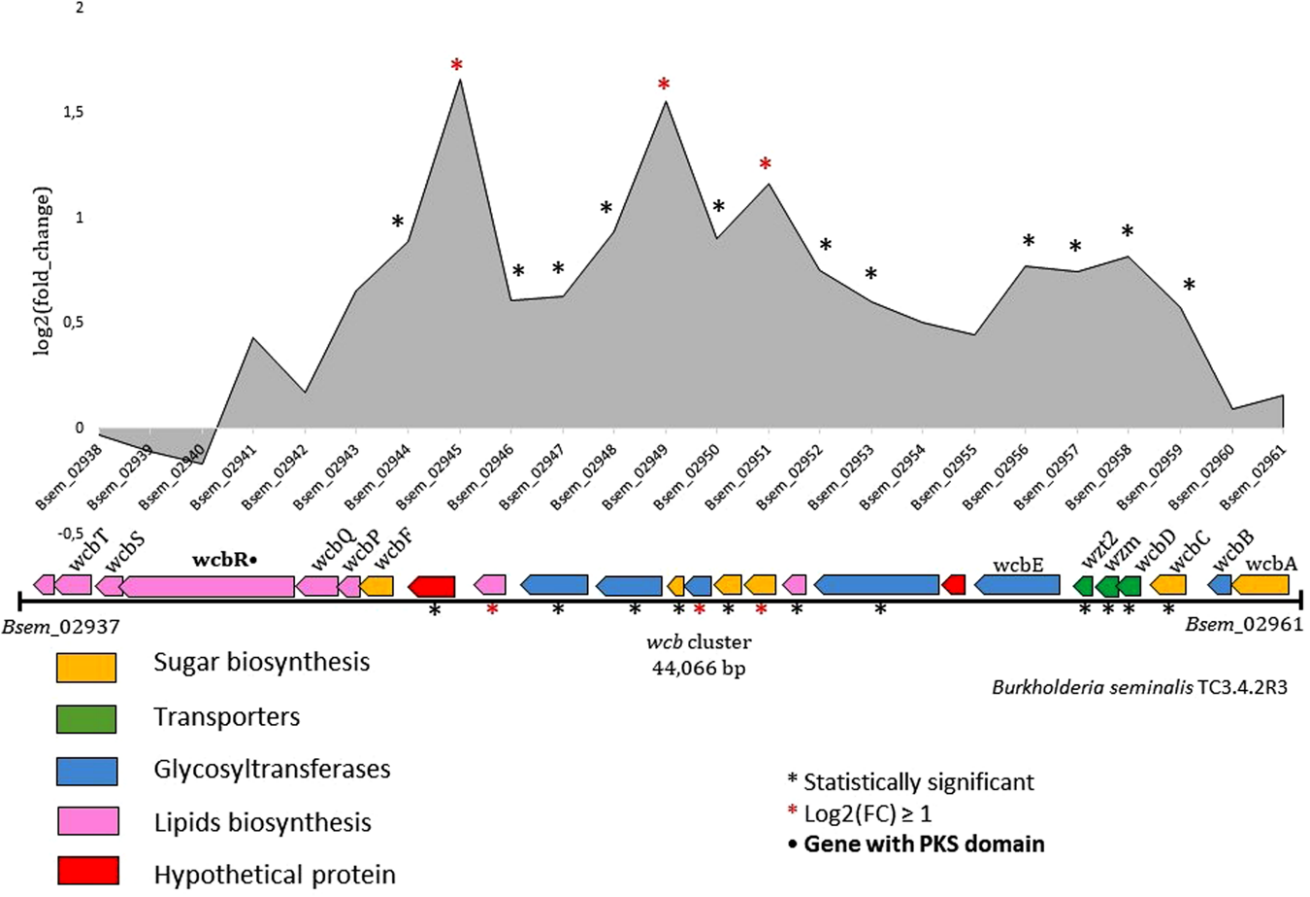
*B. seminalis wcb* cluster. Gene distribution and expression level (log2(FC)). Black asterisks indicate genes significantly expressed. Red asterisks indicate genes with log2(FC) > 1.

The capsule biosynthetic cluster *wcb* in *B. seminalis* is composed of 25 genes (*Bsem*_02937 to *Bsem*_02961) subdivided into six operons and three transcription units, of which 14 are significantly upregulated genes and three are log2(FC) > 1 (Fig. 6). *Bsem*_02945 encodes a phytanoyl-CoA dioxygenase (1.66-fold), the *Bsem*_02949 gene corresponds to a glucose-1-phosphate thymidylyltransferase (1.55-fold) and *Bsem*_02951 is a dTDP-glucose dehydratase (1.16-fold), suggesting that capsular polysaccharide production is stimulated at 37 °C.

We found that genes within the phenylacetic acid (PAA) catabolic locus were thermoregulated in *B. seminalis* (Supplementary Fig. S2). RNAseq analysis revealed that clusters *paaABIJK* and *paaNJFDK* (chromosome 1) were upregulated at 37 °C with FC values ranging from 1.11 (*Bsem*_00184) to 2.18 (*Bsem*_00388). On chromosome 2, the cluster *paaHKF* was also found to be induced (Supplementary Table S4). Furthermore, we found two genes involved in quorum sensing that were upregulated at 37 °C, *Bsem*_00682 (1.15-fold) and the autoinducer synthesis protein *Bsem*_05144 (1.18-fold).

### Secondary metabolite clusters of *B. seminalis* and temperature-dependent expression

A homology search for genes encoding known proteins with motifs involved in secondary metabolism and antibiotic production in antiSMASH revealed four putative clusters in chromosome 1, including genes encoding a nonribosomal peptide synthetase (NRPS) cluster that shares 23% amino acid similarity to cupriachelin, a terpene cluster, an arylpolyene cluster and a type I polyketide synthase (T1PKS) cluster that shares 16% amino acid similarity to a galactoglucan protein. Expression of the NRPS cluster was not regulated by temperature. The terpene cluster had 40% of the upregulated genes (1.30- to 3.21-fold), arylpolyene and T1PKS clusters, present in the *wcb* gene cluster, had 10% (1.00 to 1.60-fold) and 20% (1.16- to 1.66-fold) of upregulated genes, respectively (Supplementary Table S5).

On chromosome 2, there were nine biosynthetic clusters, including a bacteriocin, a phosphonate, an ectoine, a homoserine lactone (hser-lactone), an NRPS and four terpene clusters. The hser-lactone cluster had 20% (1.04- to 1.96-fold) of upregulated genes, followed by the terpene cluster (*Bsem*_05163-05181) with 15% (1.44- to 2.19-fold), the ectoine cluster with 10% (1.19-fold) and the terpene cluster (*Bsem*_06110-06132) with 8% (1.08 to 1.69-fold) of upregulated genes (Supplementary Table S6).

The downregulated hypothetical protein *Bsem*_04337 composed an operon of five hypothetical proteins within the bacteriocin biosynthetic cluster. Inside the NRPS cluster, pyochelin biosynthetic genes, siderophores with antimicrobial activity were found, and their expression was not differentially regulated under the test conditions. Interestingly, five genes of the core pyochelin cluster were no-reads (*Bsem*_05509 to *Bsem*_05513), while the other six genes had no variation in expression level at the evaluated temperature (Supplementary Table S7).

Chromosome 3 presented five biosynthetic clusters, including two terpenes, a ketide synthase, a pyrrolnitrin and a bacteriocin cluster. Upregulated genes ranged from 5% for the terpene cluster (*Bsem*_06661-06679) (1.58-fold) to 2% for the pyrrolnitrin cluster (1.25-fold), an antibiotic with antifungal activity (Supplementary Table S8).

Some transcriptional regulators with the ability to repress antibiotic biosynthesis were found to be upregulated at 37 °C. A gene encoding the AbrB family transcriptional regulator (*Bsem*_03584) was 2.24-fold induced at 37 °C. Furthermore, four genes belonging to the TetR family of transcriptional regulators found on chromosomes 1 and 2 were also induced, namely, *Bsem*_01769 (1.35-fold), *Bsem*_02088 (1.06-fold), *Bsem*_04768 (1.05-fold) and *Bsem*_06238 (1.06-fold).

## Discussion

**Figure 7:**
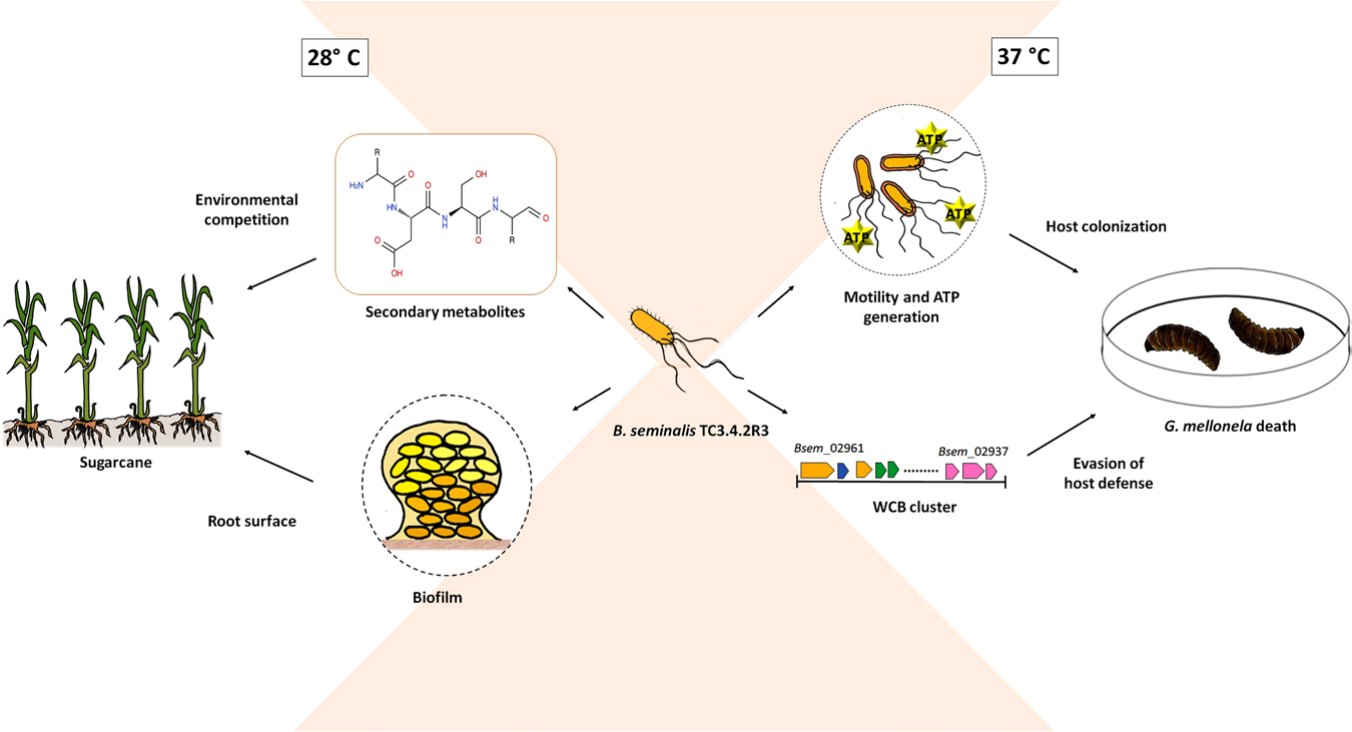
Schematic presentation of thermoregulation in *B. seminalis* TC3.4.2R3. At 28 °C, more EPS and biofilm are produced favoring the establishment of colonization. At 37 °C, energy pathways are induced, and more motility can be observed. Capsule biosynthesis is also upregulated leading to a higher virulence in larvae model.

Significant changes in gene expression in *B. seminalis* TC3.4.2R3 were observed in morphological traits comparing bacteria grown at 28 °C and 37 °C (Fig. 7). The bacteria increased biofilm formation, reduced motility, increased antimicrobial production and capsule biosynthesis and decreased virulence to *G. mellonella* at 28 °C, suggesting that the genes associated with these phenotypes could be important during interactions with the soil microbiota or during association with the plant host.

In the present study, *B. seminalis* TC3.4.2R3 produced more biofilm at 28 °C than 37 °C (Fig. 1a). Previous studies have shown that biofilm formation precedes endophytic colonization in plant tissues^35^, which should occur at temperatures less than 30 °C. In contrast, *Burkholderia* species, such as *B. pseudomallei* strain 1026b, produce more biofilm at 37 °C in comparison to 30 °C^36^, suggesting that for pathogenic bacteria, biofilm formation should be induced close to host temperature. These results suggest that for the endophytic *B. seminalis* TC3.4.2R3, the increased biofilm production at 28 °C could have a beneficial role in plant host colonization but not in virulence against *G. mellonella*.

The synthesis of EPS by *B. seminalis* TC3.4.2R3 also occurred in a temperature-dependent manner (Fig. 1b) and was greater at 28 °C than 37 °C. EPS is a major component of the biofilm matrix in many bacteria. EPS production was consistent with greater biofilm production at 28 °C. The production of EPS by *B. kururiensis* is regulated in response to growth and external conditions^37^. Similar results were observed for *B. tropica*, with biofilm formation affected in a temperature-dependent manner up to 24 h^38^. Interestingly, even though the amount of biofilm was greater at 28 °C, bacterial growth was higher at 37 °C in *B. seminalis* strain TC3.4.2R3 (Fig. 1d), indicating no association between higher growth and increased biofilm formation.

Motility can be beneficial for bacteria in terms of nutrient acquisition, translocation in specific hosts to access optimal colonization sites, avoidance of noxious agents and dispersal in the environment^39^. We found that *B. seminalis* was more motile at 37 °C than at 28 °C, suggesting that flagellar synthesis or function was thermoregulated (Fig. 1c). The flagellar apparatus presents a complex modulation, as suggested for *B. thailandensis*, which contains flagellar genes up- and downregulated at 37 °C^27^. In *B. seminalis* TC3.4.2R3, the flagellin gene *fliC* was downregulated, while the *flhD*, *flhC* and *fliL* genes were upregulated at 37 °C, suggesting differential regulation of these genes. Similar results were found in *B. pseudomallei* strain 153: more motility was observed at 37 °C than at 25 °C and at 30 °C more so than at 42 °C, although *fliC* was downregulated (−2.60-fold)^40^.

Increased flagellar activity and adaptations to warmer temperatures require a significant amount of energy to support bacterial growth and function. Interestingly, not only energy production under aerobic conditions was favoured, characterized by the over-expression of the F0/F1 ATP synthase but also anaerobic pathways, such as alcohol fermentation. Since cytochrome genes are induced, it is expected that a microaerobic environment is created because cytochromes have high affinity for oxygen, triggering oxygen depletion in *Burkholderia*^41^. For *B. seminalis* TC3.4.2R3 grown at 37 °C, genes encoding chaperons and heat-shock proteins, universal stress proteins and several genes involved in cellular detoxification were upregulated to protect and ensure cell survival, as also observed in *B. pseudomallei* strain 153 at increased temperature^40^.

Furthermore, we found a cluster of PHA biosynthesis upregulation (*Bsem*_01251 to *Bsem*_01254) at 37 °C. Polyhydroxyalkanoates (PHA) are energy-reserve polymers produced by bacteria for surviving periods of starvation in natural habitats and possibly confer a competitive advantage by allowing storage and mobilization of carbon in the face of environmental changes^41,42^. This result, in combination with the energy generation, motility and growth curve (Fig. S1), suggests that at 37 °C, *B. seminalis* TC3.4.2R3 generates more energy to sustain increased motility, but as the growth rate remains similar, the surplus energy could be stored in the form of PHA.

The increased expression of genes involved in primary metabolism, including nucleotide biosynthesis, cell division, RNA polymerases, ribosomal proteins and protein processing, indicates the high activity and energy-requiring cellular processes needed at 37 °C. Due to the accelerated activity, it is possible that cells enter a starvation period when grown at 28 °C; therefore, protein synthesis may be required during the starvation period to mediate nutrient scavenging, heat-shock response and recovery from starvation^43^. The rapid response to the availability of nutrients requires that the starved cells have an efficiently expressed transport system for nutrient uptake^44^, a result in agreement with our finding that many transmembrane and outer membrane transporters that are highly expressed at 37 °C. Moreover, the production of cell envelope structures, such as peptidoglycan, LPS and lipoproteins, was induced at 37 °C. This may be anticipated as they could confer protection to the cell during adaptation phases^45^.

Non-mammalian infection models have become particularly attractive because they are cheap, easy to assay, not limited by ethical concerns and can survive at 37 °C, thus enabling high-throughput *in vivo* screening. In this context, *G. mellonella* larvae have been used as a model organism to study bacterial pathogenicity, including members of the Bcc, because of the similarities between the innate immune systems of insects and mammals^46–48^. *B. seminalis* strains isolated from water, fruits and the rhizosphere are considered weak pathogens or non-virulent to *G. mellonella* in comparison with clinical isolates^49^. *B. seminalis* TC3.4.2R3 killed 100% of larvae in 24 h at 37 °C with 10^7^ CFU.mL^−1^, but the virulence was attenuated at 28 °C, and all larvae died in 65 h (Fig. 3). The results showed that although *B. seminalis* TC3.4.2R3 can kill wax moth larvae, this virulence is significantly increased at 37 °C. For *B. cenocepacia*, the largest number of virulence genes was induced at 37 °C in comparison to 20 °C, suggesting that temperature adaptation during host infection is associated with pathogenesis^50^.

However, in the BALB/c model, *B. seminalis* TC3.4.2R3 was not virulent, even at high concentrations (10^7^ CFU.mL^−1^), indicating that this strain is not pathogenic to mice and that the results for *G. mellonella* and mice infection models were not equivalent. Similar results were observed for *B. thailandensis*, which is avirulent in mouse infection models^51^ but is highly virulent in insects and nematoid infection models^52^. These results suggest that for some environmental strains, such as *B. seminalis* TC3.4.2R3, temperature plays a role mainly in the interaction with soil- and plant-associated microbiota, including bacteria, fungi and insects, increasing the capability of this bacterium to compete with other microorganisms and to colonize the host plant.

Many factors may be implicated in virulence, depending on the infection model. For *B. cenocepacia*, AHL-mediated quorum sensing, siderophores and LPS were found to be important virulence factors in mammals and *G. mellonella*; however, several virulence factors were host-specific for these bacteria^53,54^. In addition, it is likely that in *G. mellonella*, other virulence factors, such as flagellar motility, facilitate disease progression. Flagellar motility may be related to increased virulence in *B. pseudomallei*^55^, but pathogenic *Burkholderia* species appear to have adopted very different strategies in terms of the regulation of flagellar motility during host infection^27^.

Among the probable virulence factors, we found that capsular polysaccharide production was significantly induced at 37 °C. Capsular polysaccharides are often associated with host colonization and virulence^56^. In *B. seminalis* TC3.4.2R3, the genes *Bsem*_02944 to *Bsem*_02953, present in the central part of the WCB cluster, were upregulated at 37 °C (Fig. 6), suggesting that the capsule could be a key factor associated with virulence in *G. mellonella* but has minor roles in soil and plant interactions. A previous study showed that these genes were variably expressed in *Burkholderia*, which were associated with different lifestyles^7^.

Phenylacetic acid (PAA) is a signal molecule that could be involved in microbial interactions with the host^57^. The phenylacetic acid (PAA) catabolic pathway was shown to be required for the full pathogenicity of *B. cenocepacia* in a *Caenorhabditis elegans* nematode infection model^58^. Nevertheless, *B. seminalis* TC3.4.2R3 does not possess all genes of the PAA pathway. Clusters *paaGHIJK* and *paaABCE* are the two core functional units of the PAA catabolic pathway common in many organisms^59^. The *paaE* gene is absent in TC3.4.2R3 and has been shown to be necessary for the survival of *B. cenocepacia* in rats^60^. Therefore, the pathway for PAA degradation present on chromosomes 1 and 2 of *B. seminalis* TC3.4.2R3 may allow the partial degradation of PAA, and the accumulation of intermediates might have toxic effects on *G. mellonella* larvae, but the lack of *paaE* could explain the non-virulence in mice.

Indole-acetic acid (IAA) and phosphate solubilization are important characteristics in plant growth-promoting bacteria. IAA is an auxin that promotes cell expansion and plant growth. *B. seminalis* TC3.4.2R3 is able to produce IAA and solubilize phosphate^15^, but the expression of these genes was not thermoregulated. Additionally, in this study, *B. seminalis* had no effect on cotton and maize seed germination, regardless of the temperature.

*B. seminalis* TC3.4.2R3 presents the ability to control orchid necrosis^7^, which seems to be temperature dependent, since we observed less antimicrobial activity at 37 °C. However, the expression of the pyochelin and rhamnolipid gene clusters was not affected by temperature shift. Indeed, previous studies showed that the production of these antimicrobial compounds is induced by the presence of phytopathogenic fungi and oomycetes^16,17^. Interestingly, transcriptional factors related to repression of antibiotic biosynthesis were upregulated at 37 °C. The AbrB family transcriptional regulator inhibited bacitracin^61^ and lantibiotics^62^ biosynthesis in *Bacillus*. The TetR family of transcriptional regulators can also repress antibiotic biosynthesis, such as kanamycin^63^, auricin^64^ and the peptidyl nucleoside antibiotic, gougerotin^65^. In this work, we found four TetR genes upregulated at 37 °C, which could be associated with repression of antibiotic production in *B. seminalis* TC3.4.2R3. Additionally, less pigment was produced at 37 °C, which could also be related to a secondary metabolite.

The behaviour and pathogenesis of *B. seminalis* TC3.4.2R3 are strongly influenced by temperature. At 28 °C, the temperature that reflects conditions that the bacterium encounters in the environment, more biofilm and EPS were produced, as well as potential biocontrol capabilities such as inhibition of phytopathogen growth. In terms of colonization and plant protection, this points to optimum adaptation at 28 °C. At 37 °C, the temperature reflecting infection of a mammalian host, the bacterium was more motile and produced fewer antimicrobial compounds, suggesting that a set of genes are regulated in a temperature-specific way, which could be related to environmental/host conditions. We observed that genes that encode EPS are induced at 37 °C and that this capsular polysaccharide is an important virulence factor for larvae infection, but this bacterium failed to induce mouse death, which could be associated with the lack of genes associated with virulence. In fact, the Type 3 (T3SS), T4 (T4SS) and T6 (T6SS) secretion systems gene clusters were not completely identified in the genome of this TC3.4.2R3 strain^7^. Although *B. seminalis* TC3.4.2R3 is within the Bcc group, it does not cause disease in the murine model, which is an important consideration given its biocontrol potential in crops. However, to corroborate the biosafety of this strain as a biocontrol agent, intranasal inoculations in mice and other mammalian models should be performed to confirm the avirulence of this endophytic strain.

## Materials and Methods

### Bacterial strain and growth conditions

*B. seminalis* strain TC3.4.2R3 was isolated from inside sugarcane root tissues^15^ where it resides as an endophytic bacterium. The whole genome of the strain was previously sequenced and described^7^. *B. seminalis* was maintained at −80 °C in Luria-Bertani (LB) broth (Difco Laboratories, Sparks, USA) and 20% glycerol and was recovered on Luria-Bertani agar or broth (Difco Laboratories) with incubation for 24 h at 28 °C or 37 °C.

### Swimming motility assay

An overnight bacterial culture was harvested and resuspended in phosphate buffered saline (PBS) to an OD_600_ of 1.0. Bacterial suspensions were inoculated on the centre of LB with 0.3% w/v agar and incubated at 28 °C and 37 °C. Motility was evaluated by the appearance of growth rings around the inoculum area, and the halo diameter (mm) was measured after 24, 48 and 120 h^66^. The experiment was performed in triplicate.

### Biofilm assays

Biofilm formation was quantified using a 96-well plate and accompanying peg-lid of the MBEC (Minimum Biofilm Eradication Concentration) assay device (Innovatech, Edmonton, Canada). Wells received 150 µL bacterial suspension of *B. seminalis* standardized to 10^7^ CFU.mL^−1^ in Tryptone Soya Broth (TSB) (Difco Laboratories). Eight wells were inoculated in at least three independent experiments. The peg-lid was placed on the plate and incubated at 28 °C or 37 °C for 24 h. The peg-lid was transferred to a fresh 96-well plate containing pre-warmed TSB and incubated for more than 24 h. The peg-lid was rinsed with PBS and then baked at 60 °C for 20 minutes, followed by staining with crystal violet 0.1% (w/v) (200 µL per well) and incubation for 30 minutes at room temperature. Three wash plates containing PBS (200 µL per well) were used to rinse the pegs following staining; subsequently, the crystal violet was solubilized with 95% ethanol prior to measurement at OD_595_^67^. In addition, a growth curve was prepared. Flasks containing 100 mL of TSB were each inoculated with a bacterial suspension to reach OD_595_ equal to 0.05. Then, flasks were shaken at 150 rpm at 28 °C or 37 °C. Aliquots were removed at periods of 2 h until the stationary phase, and OD_595_ readings were measured.

### Measurement of exopolysaccharide (EPS) weight

EPS was purified from cell culture media^68^ with modifications. Briefly, *B. seminalis* was grown for 5 days at 28 °C or at 37 °C in 10 mL of mannitol medium (0.2% yeast extract and 2% mannitol). The bacterial cultures were vortexed and centrifuged at 2,300 × *g* for 10 minutes. The supernatant was transferred to new tubes, and phenol was added at a 10% final concentration and then incubated at 4 °C for 5 h and further centrifuged for 15 minutes. The water phase was collected, 4 volumes of isopropanol was added and incubated at −20 °C for 18 h. The precipitated exopolysaccharide was centrifuged at 9,100 × *g* for 15 minutes and suspended in 150 µL of distilled water. The extract was lyophilized, and the dry weight was measured.

### Antagonism against plant pathogenic fungi

An overnight *B. seminalis* culture was adjusted to 10^7^ CFU.mL^−1^ and 10 µL was cultivated for 48 h at 28 °C or at 37 °C on potato dextrose agar (PDA) (Difco Laboratories). After bacterial growth, disks of phytopathogenic fungi (6 mm) were transferred to the centre of PDA medium and incubated at 28 °C. After fungi growth, the inhibition zone was measured and compared with the control, which consisted of fungi plates without *B. seminalis*. The antagonism was evaluated against *Ceratocystis fimbriata, C. paradoxa, Colletotrichum falcatum* and *Fusarium oxysporum*. The experiment was performed in triplicate^69^. Detection of putative secondary metabolism and antibiotic genes in the three chromosomes of *B. seminalis* was performed by antiSMASH version 3.0^70^.

### Effects of *Burkholderia* on seed germination

*B. seminalis* was grown in LB broth at 28 °C or at 37 °C, shaking at 180 rpm for 48 h. Bacteria were harvested, and the absorbance was adjusted to OD_600_ 1.0 with sterile distilled water. Seeds of maize (*Zea mays*) and cotton (*Gossypium hirsutum*) were surface sterilized in 70% alcohol for 1 minute and 3% sodium hypochlorite for 1 minute and rinsed twice in sterile water. Subsequently, the seeds were immersed in the *B. seminalis* suspension at 28 °C or at 37 °C under agitation at 150 rpm for 1 h. Control seeds were treated with sterile water (untreated control). A replicate consisting of 50 seeds was placed on moist filter paper and incubated in a germination chamber at 28 °C or at 37 °C and a 12 h photoperiod for five days. During the experiment, filter paper was maintained humid, adding a suitable volume of sterile water daily and wrapping containers in transparent plastic bags. The experiment was carried out in triplicate. The mean germination percentage was calculated at the end of the experiment^71^.

### *Galleria mellonella* infection model

Larvae were obtained from LiveFoods UK and stored in woodchips at 10 °C prior to use. *B. seminalis* TC3.4.2R3 was inoculated in the hindmost larvae proleg using a 25 μL 22s-gauge gas-tight Hamilton syringe with 10 μL of 10^8^ CFU.mL^−1^ and incubated at 28 °C or at 37 °C. PBS and non-inoculated larvae were used as controls and were incubated at the same temperature as the treatments. For each strain and treatment, ten larvae were inoculated per experiment in two independent experiments. The larval survival was monitored up to 72 h^48^.

### BALB/c mouse infection model

To assess the pathogenic potential of *B. seminalis* TC3.4.2R3 in a mammalian host, six- to eight-week-old female BALB/c mice were infected. Experiments were approved by the Ethics Committee on the Use of Animals (CEUA) from University of Campinas under Registration No. 4305-1 and were conducted according to institutional standards. *B. seminalis* TC3.4.2R3 used for challenge was grown in LB broth at 37 °C under agitation (180 rpm) for 18 h. After growth, 1 mL of bacterial suspension was transferred to 50 mL of LB and incubated under the same conditions until OD_600_ 1.0 was reached. The culture was harvested for 15 minutes, and the pellet was washed with 10 mL of cold PBS. After centrifugation, the pellet was resuspended in PBS. Groups of three BALB/c mice were challenged by an intraperitoneal route with 100 µL of *B. seminalis* suspensions (10^9^, 10^8^, 10^7^, 10^6^ or 10^5^ CFU.mL^−1^). PBS was used as a control. The delivered doses of bacterial cells were then verified by plate counts on TSA. Mice were monitored for clinical signs and symptoms for 30 days.

### RNA extraction and sequencing

*B. seminalis* was grown for 48 h with shaking (180 rpm) at 28 °C or at 37 °C in TSB in triplicate. These conditions were set up based on the time (48 h) where the greatest difference in the ability to inhibit fungi between the two evaluated temperatures was observed, in which temperature was correlated with the environmental (28 °C) or clinical (37 °C) conditions.

For RNA extractions, after growth, samples were frozen with liquid nitrogen and macerated. Total RNA was extracted using the Illustra RNAspin Mini Kit (GE Healthcare, Chicago, United States) according to the manufacturer’s instructions. Samples were tested for quality in agarose gel, and 60 ng/mL of each sample was dissected with RNAstable (Biomatrica, San Diego, United States). RNA quality was evaluated by Qubit and Bioanalyzer, and only samples with intact RNA were processed. Removal of rRNA was performed using the Ribozero Bacteria (Gram-Negative) kit. The sequencing library was prepared according to the Illumina Truseq Stranded RNAseq protocol and was sequenced on the Illumina HiSeq 2000/2500 platform^46^ to generate single-end reads with 50 bp. Reads were mapped on *B. seminalis* TC3.4.2R3 chromosomes (GenBank Accession Number LAEU01000000).

### Mapping and analysis of Illumina reads

Sequence reads from each sample had rRNA and tRNA subtracted with Bowtie^72^. The reference sequences used were *E. coli* and *Burkholderia*. The resulting sequences were mapped on the *B. seminalis* TC3.4.2R3 genome (GenBank Accession Number LAEU01000000) with BWA software^73^. The Samtools package^74^ was used to convert sam into bam format files, sorted and indexed. To determine the differential expression of known transcripts, the resulting aligned reads were analysed by TopHat^75^ and Cuffdiff package^76^. Transcripts mapping to the *B. seminalis* TC3.4.2R3 genome with a *P*-value of ≤0.05 were considered differentially expressed transcripts, and those with log2(FC) above 1 were analysed further. Relative expression graphs and tables were assembled using the R program and the CummeRbund library^77^. Volcano plots were made in R. Functional and Gene Ontology annotations of the genome were performed with Blast2GO software^78^. Furthermore, transcripts were analysed by dbCAN (carbohydrate-active enzyme annotation)^79^, SignalP for the presence and location of signal peptide^80^, EffectiveDB for prediction of bacterial protein secretion^81^ and PSORTb for bacterial protein subcellular localization prediction^82^.

### Statistical analysis

All *in vitro* assays were performed at least in triplicate, with subsequent statistical analysis by one-way ANOVA with Tukey’s test (GraphPad Prism 7.0) and multiple comparisons. For growth curves, linear regression was used to calculate line equations, and curves were compared by the F test. *P* < 0.05 was deemed statistically significant. A Kaplan-Meier survival plot with log-rank (Mantel-Cox) test and Bonferroni correction (GraphPad Prism 7.0) was used to compare survival of *G. mellonella*.

## Supplementary information

Supplementary Data
