# Supplementary material for: Environmental interactions are regulated by temperature in Burkholderia seminalis TC3.4.2R3
Source: Sci Rep. 2019 Apr 2;9:5486. doi: 10.1038/s41598-019-41778-x (PMC6445077; doi:10.1038/s41598-019-41778-x)
Supplement: Supplementary file 1 — Supplementary Data [file 41598_2019_41778_MOESM1_ESM.pdf]

Supplementary information

**Environmental interactions are regulated by temperature in *Burkholderia seminalis* TC3.4.2R3.**

Priscila Jane Romano de Oliveira Gonçalves, Carmen C. Denman Hume, Almir José Ferreira, Sarina Tsui, Marcelo Brocchi, Brendan W. Wren, Welington Luiz Araujo.

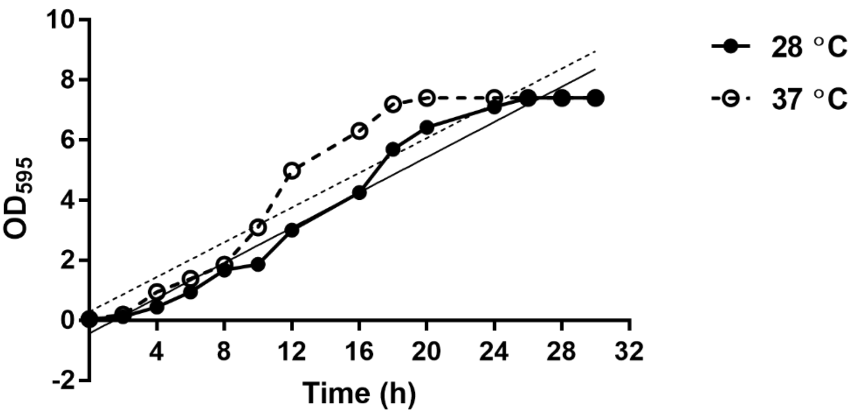


**Supplementary Figure S1.** Growth curve of *B. seminalis* TC3.4.2R3 at 28ºC and 37ºC, measured by optical density at 595 nm ($OD_{595}$), in TSB is shown over 30h.

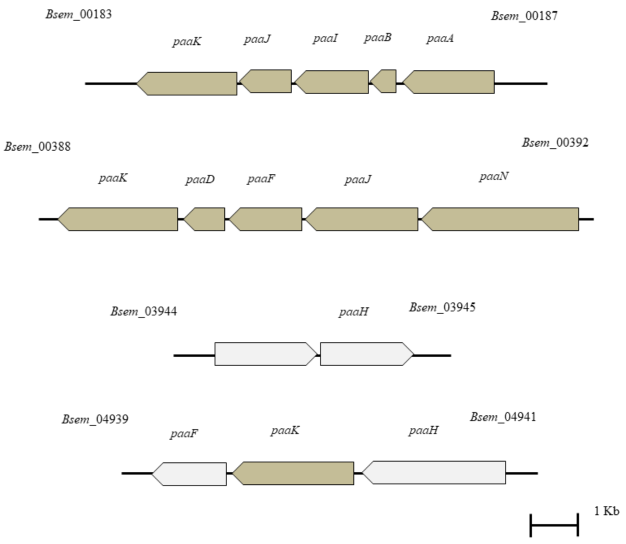


**Supplementary Figure S2.** Genetic organization of the PAA catabolic gene clusters in *B. seminalis* TC3.4.2R3. Gray arrows indicate upregulated genes at 37ºC (log2(FC) > 1). *paa*ABIJK and *paa*NJFDK are located on the chromosome 1 and *paa*HKF and *paa*H is located on chromosome 2.

**Supplementary Table S1** – Percentages of seed germination at different temperatures. Values correspond to averages of triplicates following by standard deviation.

| | 28ºC | | 37ºC | |
|---|---|---|---|---|
| | TC3.4.2R3 | Control | TC3.4.2R3 | Control |
| Maize | 90.7 ± 4.16 | 86.7 ± 3.06 | 79.3 ± 12.06 | 81.3 ± 12.06 |
| Cotton | 76.0 ± 9.17 | 78.0 ± 10.39 | 88.7 ± 7.57 | 86.7 ± 4.62 |

**Supplementary Table S2**. Down-regulated genes in *Burkholderia seminalis* TC3.4.2R3 genome at 37 ºC with counted mapped reads.

| **Gene ID** | **28 ºC** | **37 ºC** | **Fold change** | **Gene function** |
|---|---|---|---|---|
| *Bsem*_00099 | 940.66 | 209.33 | -2.17 | Flagellin FliC |
| *Bsem*_01205 | 455.52 | 209.92 | -1.12 | hypothetical protein |
| *Bsem*_04240 | 7633.46 | 2116.99 | -1.85 | hypothetical protein |
| *Bsem*_04337 | 198.04 | 74.78 | -1.41 | hypothetical protein |
| *Bsem*_04629 | 8348.66 | 2947.38 | -1.50 | hypothetical protein |
| *Bsem*_04631 | 14242.20 | 1022.22 | -3.80 | hypothetical protein |

**Supplementary Table S3.** Counts of mapped reads of *B. seminalis* TC3.2.3R4 involved in flagella biosynthesis and assembly.

| **Gene ID** | **28ºC** | **37ºC** | **Fold change** | **Gene function** |
|---|---|---|---|---|
| *Bsem*_00108 | 536.53 | 2436.35 | 2.18 | transcriptional activator FlhD |
| *Bsem*_00109 | 242.39 | 1058.83 | 2.13 | transcriptional activator FlhC |
| *Bsem*_02663 | 565.27 | 2338.31 | 2.05 | OmpA/MotB domain-containing protein |
| *Bsem*_02929 | 331.46 | 868.51 | 1.39 | OmpA/MotB |
| *Bsem*_03217 | 302.24 | 600.37 | 1.00 | flagellar basal body-associated protein FliL |

**Supplementary Table S4.** Counts of mapped reads of *B. seminalis* TC3.2.3R4 transcripts involved in amino acid biosynthesis and metabolism.

| Gene ID | 28°C | 37°C | Fold change | Gene function |
|---|---|---|---|---|
| *Bsem*_00183 | 314.47 | 886.89 | 1.50 | phenylacetate-CoA oxygenase/reductase subunit PaaK |
| *Bsem*_00184 | 527.63 | 1140.02 | 1.11 | phenylacetate-CoA oxygenase subunit PaaJ |
| *Bsem*_00185 | 483.61 | 1330.35 | 1.46 | phenylacetate-CoA oxygenase subunit PaaI |
| *Bsem*_00186 | 1387.26 | 3656.83 | 1.40 | phenylacetate-CoA oxygenase subunit PaaB |
| *Bsem*_00187 | 606.20 | 1720.30 | 1.50 | phenylacetate-CoA oxygenase subunit PaaA |
| *Bsem*_00388 | 145.08 | 660.23 | 2.19 | phenylacetate-CoA ligase |
| *Bsem*_00389 | 280.61 | 847.79 | 1.60 | phenylacetic acid degradation protein PaaD |
| *Bsem*_00390 | 289.72 | 854.77 | 1.56 | enoyl-CoA hydratase |
| *Bsem*_00391 | 264.11 | 724.90 | 1.46 | acetyl-CoA acetyltransferase |
| *Bsem*_00392 | 379.55 | 1384.52 | 1.87 | phenylacetic acid degradation protein paaN2 |
| *Bsem*_00393 | 227.92 | 477.83 | 1.07 | enoyl-CoA hydratase |
| *Bsem*_04940 | 236.36 | 620.40 | 1.39 | phenylacetate-CoA ligase |

**Supplementary Table S5.** Upregulated genes (log2(FC) >1) in biosynthetic genes clusters located on chromosome 1. FC: fold change.

| Metabolite | Sequence size (bp) | Total number of genes | Gene ID | Product | FC |
|---|---|---|---|---|---|
| Terpene | 21046 | 20 | *Bsem*_01401 | Hypothetical protein | 1.36 |
| | | | *Bsem*_01403 | Hypothetical protein | 2.89 |
| | | | *Bsem*_01405 | Domain of unknown function DUF1828 | 1.30 |
| | | | *Bsem*_01407 | Sulfotransferase domain protein | 1.76 |
| | | | *Bsem*_01416 | Alkyl hydroperoxide reductase/ Thiol specific antioxidant | 3.21 |
| Arylpolyene | 41250 | 37 | *Bsem*_02248 | Acetoacetyl-CoA reductase | 1.24 |
| | | | *Bsem*_02249 | Acetyl-CoA acetyltransferase | 1.60 |
| | | | *Bsem*_02251 | putative ParA family protein | 1.19 |
| | | | *Bsem*_02254 | Single-stranded DNA-binding protein | 1.00 |
| T1 PKS (Galactoglucan) | 47628 | 41 | *Bsem*_02913 | Hypothetical protein | 1.59 |
| | | | *Bsem*_02922 | Serine hydroxymethyltransf erase | 1.54 |
| | | | *Bsem*_02929 | OmpA/MotB | 1.39 |
| | | | *Bsem*_02930 | Hypothetical protein | 1.28 |
| | | | *Bsem*_02936 | Adenylyl-sulfate kinase | 1.61 |
| | | | *Bsem*_02945 | Phytanoyl-CoA dioxygenase | 1.66 |
| | | | *Bsem*_02949 | Glucose-1-phosphate thymidylyltransferas e | 1.55 |
| | | | *Bsem*_02951 | dTDP-glucose 4,2C6-dehydratase | 1.16 |

**Supplementary Table S6.** Upregulated genes (log2(FC) >1) in biosynthetic genes clusters located on chromosome 2. FC: fold change.

| Metabolite | Sequence size (bp) | Total number of genes | Gene ID | Product | FC |
|---|---|---|---|---|---|
| Ectoine | 10398 | 10 | *Bsem*_04974 | Hypothetical protein | 1.19 |
| Hserlactone | 20608 | 24 | *Bsem*_05144 | Autoinducer synthesis protein | 1.18 |
| | | | *Bsem*_05150 | signal-transduction protein | 1.32 |
| | | | *Bsem*_05151 | Hypothetical protein | 1.04 |
| | | | *Bsem*_05152 | GreA/GreB family elongation factor | 1.24 |
| | | | *Bsem*_05153 | Porin | 1.96 |
| Terpene | 21034 | 19 | *Bsem*_05170 | FAD-dependent pyridine nucleotide-disulfide oxidoreductase | 2.19 |
| | | | *Bsem*_05171 | Hypothetical protein | 1.66 |
| | | | *Bsem*_05172 | Hypothetical protein | 1.44 |
| Terpene | 24101 | 23 | *Bsem*_06116 | Toluene tolerance | 1.08 |
| | | | *Bsem*_06132 | cobyrinic acid - diamide synthase | 1.69 |

**Supplementary Table S7.** Counts of mapped reads of *B. seminalis* TC3.2.3R4 involved in pyochelin biosynthesis.

| Gene ID | 28°C | 37°C | Fold change | Gene function |
|---|---|---|---|---|
| *Bsem*_05508 | 547.20 | 991.44 | 0.86 | TonB-dependent siderophore receptor |
| *Bsem*_05509 | 0 | 0 | 0.00 | ABC transporter |
| *Bsem*_05510 | 0 | 0 | 0.00 | ABC transporter related |
| *Bsem*_05511 | 0 | 0 | 0.00 | thiazolinyl imide reductase |
| *Bsem*_05512 | 0 | 0 | 0.00 | amino acid adenylation |
| *Bsem*_05513 | 0 | 0 | 0.00 | amino acid adenylation |
| *Bsem*_05514 | 327.62 | 516.52 | 0.66 | transcriptional regulator PchR |
| *Bsem*_05515 | 916.79 | 1021.36 | 0.16 | AMP-dependent synthetase and ligase |
| *Bsem*_05516 | 592.78 | 834.51 | 0.49 | thioesterase |
| *Bsem*_05517 | 4018.93 | 5030.59 | 0.32 | isochorismate-pyruvate lyase |
| *Bsem*_05518 | 619.75 | 829.08 | 0.42 | salicylate biosynthesis isochorismate synthase |

**Supplementary Table S8.** Upregulated genes (log2(FC) >1) in biosynthetic genes clusters located on chromosome 3. FC: fold change.

| Metabolite | Sequence size (bp) | Total number of genes | Gene ID | Product | FC |
|---|---|---|---|---|---|
| Other KS | 45966 | 31 | *Bsem*_06618 | Hypothetical protein | 1.58 |
| Terpene | 21082 | 18 | *Bsem*_06668 | methyltransferase type 12 | 1.15 |
| Pyrrolnitrin | 41091 | 35 | *Bsem*_06816 | putative transmembrane protein | 1.25 |
